# Change in healthcare utilization before and after COVID‐19 using data from 1.5 million individuals

**DOI:** 10.1111/joim.70051

**Published:** 2025-11-27

**Authors:** Maria Bygdell, Erik Bülow, Simon B. Larsson, Robert Sigström, Huiqi Li, Jari Martikainen, Ailiana Santosa, Lisa Lundberg‐Morris, Susannah Leach, Magnus Gisslén, Carl Bonander, Jörgen Månsson, Kristoffer Strålin, Fredrik Nyberg

**Affiliations:** ^1^ Department of Internal Medicine and Clinical Nutrition, Institute of Medicine, Sahlgrenska Academy University of Gothenburg Gothenburg Sweden; ^2^ School of Public Health and Community Medicine, Institute of Medicine, Sahlgrenska Academy University of Gothenburg Gothenburg Sweden; ^3^ Department of Infectious Diseases Institute of Biomedicine at the Sahlgrenska Academy University of Gothenburg Gothenburg Sweden; ^4^ Department of Addictions Sahlgrenska University Hospital, Region Västra Götaland Gothenburg Sweden; ^5^ Department of Psychiatry and Neurochemistry, Institute of Neuroscience and Physiology, Sahlgrenska Academy University of Gothenburg Gothenburg Sweden; ^6^ Department of Psychiatry for Affective Disorders Sahlgrenska University Hospital, Region Västra Götaland Gothenburg Sweden; ^7^ Bioinformatics and Data Centre, The Sahlgrenska Academy University of Gothenburg Gothenburg Sweden; ^8^ Department of Drug Treatment Sahlgrenska University Hospital, Region Västra Götaland Gothenburg Sweden; ^9^ Department of Microbiology and Immunology, Institute of Biomedicine, Sahlgrenska Academy University of Gothenburg Gothenburg Sweden; ^10^ Department of Infectious Diseases, Institute of Biomedicine, Sahlgrenska Academy University of Gothenburg Gothenburg Sweden; ^11^ Department of Infectious Diseases Sahlgrenska University Hospital, Region Västra Götaland Gothenburg Sweden; ^12^ Public Health Agency of Sweden Solna Sweden; ^13^ Centre for Societal Risk Research Karlstad University Karlstad Sweden; ^14^ Department of Infectious Diseases Karolinska University Hospital Stockholm Sweden; ^15^ Department of Medicine Huddinge Karolinska Institute Stockholm Sweden

**Keywords:** COVID‐19, difference‐in‐difference, healthcare utilization, long‐term effects

## Abstract

**Background and objective:**

Post‐infectious sequelae can increase burden on healthcare systems. We aimed to assess the long‐term effect of COVID‐19 on healthcare utilization across all levels of care.

**Methods:**

In this register‐based cohort study, we included all adult (≥18 years) residents in Sweden's two largest counties with a registered COVID‐19 index date between 31 January 2020 and 9 February 2022. Each exposed individual was matched 1:1 to a control without registered COVID‐19 on index date based on gender, birth year, vaccination status and the change in number of healthcare contacts between 2018 and 2019. We counted the number of healthcare contacts across all levels of care during the pre‐index (13–1 months) and post‐index (4–15 months) full‐year periods. A difference‐in‐difference (DID) analysis was used to assess changes in the number of healthcare contacts and specific diagnoses, between each individual's pre‐ and post‐periods, as well as comparing individuals with and without COVID‐19.

**Results:**

The study included 753,905 matched pairs, comprising 1,415,432 unique individuals. Trends in healthcare contacts were parallel between the matched groups prior to the index date. The DID analysis revealed a mean increase of 0.33 (95%CI 0.30–0.36) healthcare contacts following COVID‐19, mainly observed from a smaller proportion of the population (5%) and by contacts with primary healthcare. The largest diagnosis‐specific difference was observed for reactions to severe stress (0.02, 0.01–0.03). The estimate varied across gender, acute COVID‐19 severity, virus variant period and vaccination status.

**Conclusion:**

This study demonstrates increased healthcare utilization after COVID‐19 in a smaller proportion of the population.

## Graphical abstract

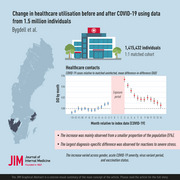

## Introduction

Early in 2020, COVID‐19 was declared a global pandemic, and by December 2024, almost 800 million COVID‐19 cases including 7 million deaths had been reported to the World Health Organization (WHO) [1]. A hallmark of severe acute respiratory syndrome (SARS)‐CoV‐2 infection is its wide range of disease severity, with notable variation also across different viral variant strains [2]. Most patients have experienced mild symptoms, some have remained asymptomatic, yet for a minority of patients, especially in 2020–2021, COVID‐19 was a life‐threatening condition requiring hospitalization or intensive care [3, 4]. Already a few months into the pandemic, reports emerged describing persistent symptoms among recovered hospitalized and non‐hospitalized COVID‐19 patients [5, 6, 7]. A range of persistent or newly emerging symptoms have been reported in patients experiencing long‐term effects, including fatigue, dyspnoea, cardiovascular abnormalities (e.g., myocardial inflammation and postural orthostatic tachycardia syndrome), cognitive dysfunction and mental health problems and olfactory and gustatory dysfunctions [8]. The concept of post‐infectious sequelae has been previously described for several pathogens [9] including influenza [10], as well as SARS and Middle East respiratory syndrome (MERS) [11]. As SARS‐CoV‐2 belongs to the same coronavirus family as SARS and MERS, its long‐term effects may share similarities. However, although SARS and MERS collectively affected only around 12,000 individuals during the outbreaks in 2003 and 2012, COVID‐19 has had a far greater impact. In Sweden alone, more than 2.7 million cases had been reported to WHO as of December 2024 although almost the whole population is expected to have been infected at least once [1]. Given the scale of the pandemic, post‐COVID‐19 sequelae pose not only a significant challenge for individuals but also a substantial burden on society and healthcare systems.

In addition to the large number of people affected by COVID‐19 and consequently at risk for post‐infectious sequelae, the length of the recovery period influences the burden on healthcare systems. Currently, there is still no conclusive evidence on the prognosis and recovery of post‐infectious sequelae from COVID‐19, although a Norwegian study from 2021 that evaluated primary healthcare utilization among SARS‐CoV‐2 positive patients with mild disease before and after COVID‐19 compared to test‐negative controls [12] found a short‐term increase in healthcare utilization 2–3 months after a positive test, which subsequently declined. This finding suggests that most patients with persistent symptoms may recover within the first 3 months after the acute state. However, these results contrast with other studies that examined self‐reported experiences of long‐term symptoms after COVID‐19. For example, a US study found that 1 in 5 adults reported not having fully recovered within 90 days of acute infection [13].

In the present study, we aimed to assess the potential elevated healthcare burden across all levels of healthcare following COVID‐19. Using a difference‐in‐difference (DID) design, we analysed data from a comprehensive database covering specialist inpatient care, specialist physician outpatient care and primary healthcare for all individuals in the two largest counties in Sweden (approximately 40% of the Swedish population) [14].

## Methods

### Study design and data sources

The Swedish COVID‐19 Investigation for Future Insights—a Population Epidemiology Approach using Register Linkage (SCIFI‐PEARL) project is a register‐based cohort study in Sweden with comprehensive data collected from a unique linkage of several Swedish national, regional and quality registers using the Swedish personal identity number [15, 16]. The present study is part of the SCIFI‐PEARL research effort and included data from the National Register of Notifiable Diseases (SmiNet, [17]) the National Vaccination Register (NVR), the National Patient Register (NPR, [18]) regional primary healthcare databases in Stockholm (VAL) and Västra Götaland (VEGA), the Swedish Intensive Care Register (SIR) and the Longitudinal Integrated Database for Health Insurance and Labour Market Studies (LISA) [19]. SmiNet includes all positive SARS‐CoV‐2 polymerase chain reaction (PCR) test results, NVR comprises all COVID‐19 vaccination doses, NPR covers all inpatient and specialist physician outpatient healthcare visits, [18, 20] the VAL and VEGA databases include all public and most private primary healthcare contacts in Stockholm and Västra Götaland county (the two largest counties in Sweden with nearly 40% of the Swedish population (14)), SIR comprises all intensive care stays, and the LISA database covers demographic and socioeconomic data for all Swedish residents. The study was approved by the Swedish Ethical Review Authority (Dnr: 2020‐01800 with several amendments).

### Study population

The exposed study population included all adults (≥18 years of age on 1 January 2018) residing in Stockholm or Västra Götaland county during the study period (obtained from the LISA database) who had a registered COVID‐19 between the start of study inclusion (31 January 2020, the date of the first COVID‐19 case in Sweden) and the end of study inclusion (9 February 2022, the last date of full population testing in Sweden) (Fig. S1). Their index date was set to the date of the registered COVID‐19 (see below). In addition, we used risk set matching [21] to create an unexposed comparison group matched 1:1 on birth year, gender, vaccination status before index (≥1 dose or 0 doses) and the change in number of healthcare contacts between the two pre‐index years 2018 and 2019 (exact if −20 to 20, otherwise categorized in bins of approximately equal sizes). Individuals in the unexposed group had no registered COVID‐19 up until the index date of their matched COVID‐19‐exposed counterpart. Unexposed individuals were randomly selected without replacement (i.e., one unexposed individual could not be the control for multiple exposed individuals). However, an exposed individual could also serve as an unexposed control prior to his or her index date.

### Exposure

COVID‐19 was defined as a first positive SARS‐CoV‐2 PCR test result recorded in SmiNet and/or a diagnosis of COVID‐19 (International Classification of Diseases version 10 Swedish edition (ICD‐10‐SE) U07.1 or U07.2, as either main or secondary diagnosis) in NPR/VEGA/VAL. The first registration of any of these was defined as the index date for each exposed individual. Each unexposed individual was assigned the same index date as his or her exposed match. During the first 3 months following the index date, COVID‐19 severity was categorized as either hospitalized (requiring intensive care or non‐intensive inpatient care) or not hospitalized (requiring neither intensive nor inpatient care) for the acute COVID‐19. This categorization was based on main diagnoses of COVID‐19 from NPR's inpatient registrations or SIR.

### Outcome and follow‐up

For each matched pair, we counted the number of days with initiated healthcare contacts (including telephone contacts, but purely administrative calls, e.g., booking, cancellations or general inquires, were not counted) with primary healthcare, outpatient hospital specialist physician care and inpatient hospital specialist care both before index date and during follow‐up. More than one contact initiated on the same day was counted as one contact. For inpatient care, each inpatient stay within a specific clinic was counted as one contact, recorded on the date of admission. The number of healthcare contacts before COVID‐19 was assessed in three full‐year periods: from 1 January 2018 to 31 December 2018, from 1 January 2019 to 31 December 2019 and from 13 months before index date until 1 month before index date. The follow‐up period or full‐year started after 3 months from index date (for matched pairs that were still alive and resident) and lasted until the earliest of: SARS‐CoV‐2 infection or reinfection, moving out of Stockholm or Västra Götaland county, emigration, death, 15 months from index, or end of follow‐up (31 May 2024). The number of healthcare contacts after COVID‐19 was counted from start of follow‐up (3 months from index date) until end of follow‐up (maximum of 15 months from index). If end of follow‐up was reached before the end of the defined 1‐year post‐index period but occurred later than at least 3 months of follow‐up (e.g., after 6 months from index date), the number of contacts was extrapolated throughout the whole post‐period as the number of contacts observed before termination of follow‐up divided by the fraction of time at risk. If follow‐up was shorter than 3 months (e.g., before 6 months from index date), the observed number of contacts was retained (see Supporting Information section for details and sensitivity analyses of this approach).

Diagnosis codes (first three positions of ICD‐10‐SE) for each healthcare contact were retrieved from NPR/VEGA/VAL, based on main/first diagnoses (if the main/first diagnosis code was COVID‐19 then the secondary diagnosis code was used instead). If multiple healthcare contacts occurred on the same day, all main diagnosis codes were used.

### Covariates

Sociodemographic data, including age, gender, country of birth, education and employment, were obtained from the LISA database. Age at the index date was categorized into five groups (18–34, 35–44, 45–54, 55–64 and ≥65 years of age). Country of birth was classified as Sweden, not Sweden and unknown. Education was categorized into primary (<10 years), secondary (10–12 years) and tertiary school level (>12 years), and unknown. Employment was categorized as employed or unemployed. The Charlson comorbidity index (CCI) was calculated based on recorded diagnoses before the inclusion date. We categorized the index date into distinct time periods corresponding to the dominant virus variant at that time. In Sweden, wild‐type variants dominated from February 2020 to January 2021, followed by the Alpha variant from February 2021 to June 2021, Delta variant from July 2021 to December 2021 and Omicron variant from January 2022 to the end of inclusion on 9 February 2022.

### Subgroup analyses

We performed four subgroup analyses, stratifying the total population according to gender, acute disease severity, vaccination status pre‐index and time periods corresponding to dominating virus variants.

### Sensitivity analysis

We performed a sensitivity analysis where we did not terminate follow‐up of exposed individuals at reinfection.

### Statistical analyses

Descriptive statistics are presented as median and interquartile range (IQR) and mean and standard deviation for continuous variables and as count and percentages for categorical variables. Standardized mean differences (SMD) were calculated for sociodemographic variables, CCI and inpatient/outpatient healthcare variables. These were used to validate sample balance after matching and to quantify potential differences among the exposed and unexposed groups. An SMD less than 0.1 was considered to indicate negligible imbalance between groups. Healthcare contacts were analysed across three pre‐index periods and one post‐index period. The number of healthcare contacts was reported during 1‐year periods and categorized as follows: any healthcare contact, primary care telephone consultations, primary care other, outpatient hospital specialist physician care and inpatient hospital specialist care.

We estimated the effect of COVID‐19 on the number of healthcare contacts by comparing the change from before to after index date in the exposed individuals compared to their individual unexposed matches using a DID approach [22]. DID is a method that in the present study compares the number of healthcare contacts after an event to before the event as well as compared to an unexposed group to eliminate underlying trends. Therefore, the method takes any time‐invariant confounding and underlying trend into account, assuming the latter would have been parallel between the two groups had the COVID‐19 exposure never occurred. We assessed the assumption of parallel trends in the pre‐period by visualizing the mean number of healthcare contacts for exposed and unexposed individuals each month relative to the index date. We computed DID estimates with robust errors using a paired estimation procedure that accounts for dependency due to repeated measures and the matching process (see Supporting Information section for details). DID estimates were calculated for number of contacts, as well as for any specific diagnosis codes reported. *p*‐values for the diagnosis code‐specific tests were adjusted for multiple comparisons using a false discovery rate of 0.05. We also applied a quantile DID (QDID) analysis to assess distributional effects (see Supporting information section for details).

We used SAS v. 9.4 (SAS Institute Inc., 2013) and R version 4.4.1 (R Core Team 2024) for all statistical analyses.

## Results

During the 4–15 months study period, 753,905 adult residents of Stockholm and Västra Götaland counties had their first recorded COVID‐19 (Fig. S1). In total, the matched study included 1,415,432 unique individuals, as some individuals appeared in two matched groups—initially as a matched unexposed individual prior to their infection date and later as exposed after contracting COVID‐19. Among these exposed individuals, 739,670 (98%) had at least 3 months of follow‐up (6 months from the index date) and 685,524 (91%) had 1 year of follow‐up (15 months from the index date). Among the unexposed, 735,405 (98%) had at least 3 months of follow‐up (6 months from index date) and 662,454 (88%) had 1 year of follow‐up (15 months from index date) (Figs. S1 and S2). The unexposed group had somewhat shorter follow‐up than the exposed group mainly because of termination of follow‐up due to contracting the infection.

Exposed and unexposed individuals were completely balanced regarding the matching variables. Most pre‐index demographic characteristics and comorbidities were similar between the groups (SMD < 0.1), but the exposed individuals were to a higher extent employed (SMD  =  0.15, Table 1). In the exposed group, 28,153 (3.7%) were hospitalized due to their COVID‐19 (2373 required intensive care and 25,780 received non‐intensive inpatient care), and 725,752 (96%) were not hospitalized. Most of the exposed individuals had an index date during the wild‐type variant period (33%) or during the Omicron variant‐dominated period (35%), and only 10% had an index date during the Delta variant‐dominated period.

**Table 1:** Descriptive statistics of the study population including all adult residents in the two largest counties in Sweden in 2020, by exposure status (registered COVID‐19 or no COVID‐19).

Characteristic	Exposed (COVID‐19) *n* = 753,905	Unexposed (no COVID‐19) *n* = 753,905	Standardized mean difference
Gender			0.00
Men	350,135 (46%)	350,135 (46%)	
Women	403,770 (54%)	403,770 (54%)	
Age (years) median (q1–q3)	44 (33–55)	44 (33–55)	0.00
Age group at index^a^			0.02
18–34 years	210,389 (28%)	211,316 (28%)	
35–44 years	182,047 (24%)	181,022 (24%)	
45–54 years	170,064 (23%)	168,938 (22%)	
55–64 years	112,094 (15%)	108,600 (14%)	
≥65 years	79,311 (11%)	84,029 (11%)	
Country of birth			0.02
Sweden	562,897 (75%)	554,969 (74%)	
Not Sweden	190,944 (25%)	198,861 (26%)	
Unknown	64 (<0.01%)	75 (<0.01%)	
Highest attained education in 2019			0.08
Primary school	79,027 (10%)	90,661 (12%)	
Secondary school	305,753 (41%)	297,996 (40%)	
Tertiary school	360,608 (48%)	351,131 (47%)	
Unknown	8517 (1.1%)	14,117 (1.9%)	
Employment in 2019			0.15
Employed	659,360 (87%)	619,725 (82%)	
Unemployed	94,545 (13%)	134,180 (18%)	
Pre‐index CCI			0.02
0	694,375 (92%)	697,831 (93%)	
1	25,024 (3.3%)	23,264 (3.1%)	
2	24,393 (3.2%)	24,040 (3.2%)	
≥3	10,113 (1.3%)	8770 (1.2%)	
Difference in number of healthcare contacts between 2018 and 2019, median (q1, q3)	0 (−2, 2)	0 (−2, 2)	0.00
Difference in number of healthcare contacts between 2018 and 2019			0.00
≤−11	35,151 (4.7%)	35,151 (4.7%)	
−10 to −4	91,183 (12%)	91,183 (12%)	
−3 to −1	164,228 (22%)	164,228 (22%)	
0	151,729 (20%)	151,729 (20%)	
1–3	164,534 (22%)	164,534 (22%)	
4–10	101,847 (14%)	101,847 (14%)	
≥11	45,233 (6.0%)	45,233 (6.0%)	
COVID‐19‐related characteristics			
Vaccinated before index (any dose)	300,727 (40%)	300,727 (40%)	0.00
Index date in time period corresponding to predominant virus variant			
Wild‐type variant period	249,482 (33%)		
Alpha variant period	165,382 (22%)		
Delta variant period	76,713 (10%)		
Omicron variant period	262,328 (35%)		
Severity of the COVID‐19 acute phase			
Not hospitalized	725,752 (96%)		
Hospitalized—non‐ICU	25,780 (3.4%)		
Hospitalized—ICU	2373 (0.3%)		

Abbreviations: CCI, Charlson Comorbidity Index; ICU, intensive care unit; q1, quartile 1; q3, quartile 3.

^a^Matched on birth year, age calculated at index.

### Healthcare contacts before and after COVID‐19

**Fig. 1:**
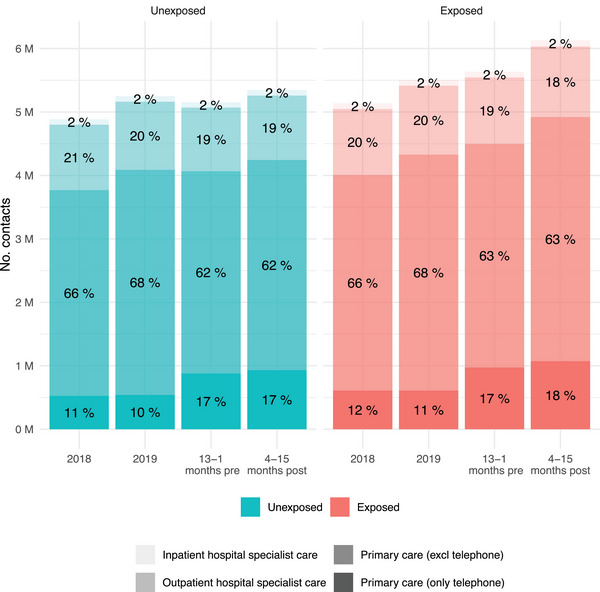
Number and type of healthcare contacts for the exposed (COVID‐19) and unexposed (no COVID‐19) groups, in time periods before COVID‐19 (2018, 2019, 13–1 months) and after COVID‐19 (4–15 months). The study population includes all adult residents in the two largest counties in Sweden in 2020.

**Fig. 2:**
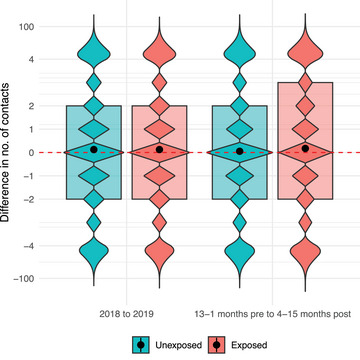
Number of healthcare contacts in the exposed (COVID‐19) and the unexposed (no COVID‐19) group before the pandemic (difference between 2018 and 2019) and in the pandemic (13–1 month before index and 4–15 months after index). Violin plot, black dots indicate mean. The y‐axis shows discrete values, and beyond 4 the scale is logarithmic. The study population includes all adult residents in the two largest counties in Sweden in 2020.

**Fig. 3:**
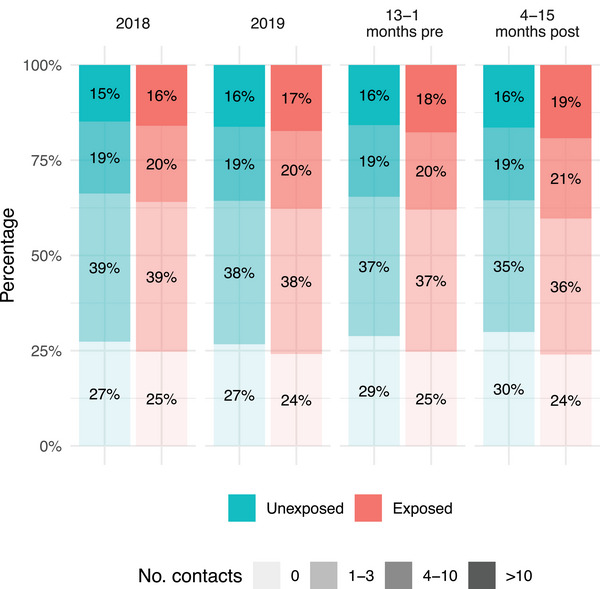
Proportion of individuals in categories of number of healthcare contacts before COVID‐19 (2018, 2019, 13–1 month pre‐index) and after COVID‐19 (4–15 months post‐index) for the exposed (registered COVID‐19) and unexposed (no COVID‐19) groups. The study population includes all adult residents in the two largest counties in Sweden in 2020.

During the year after COVID‐19 diagnosis (4–15 months from index date) compared to the year before (13–1 months before index date), there was an increase in the total number of healthcare contacts in both the unexposed and the exposed groups, primarily for primary healthcare contacts (Fig. 1). The mean number of healthcare contacts was lower in the unexposed group (5.8 contacts) compared to the exposed group (6.7 contacts) in the post‐index time period as well as in pre‐index time period (unexposed: 5.6, exposed: 6.2) (Table 2 and Fig. 2). The proportion of individuals with zero contacts increased both in the unexposed and the exposed group in the pre‐index time period compared to 2018 and 2019 (probably due to adherence to national contact restrictions during the pandemic). In the post‐index period, the proportion of exposed individuals with frequent healthcare contacts (4–10 visits and >10 visits) increased compared to the pre‐index period (Fig. 3 and Fig. S3).

**Table 2:** Number of healthcare contacts among matched adult residents (n  =  1,415,432) in the two largest counties in Sweden in 2020, in the different study time periods (three time periods before index date (defined as date of registered COVID‐19 for exposed, or matched date for unexposed) and one time period after index date), by exposure status in the study population.

	Exposed (COVID‐19)		Unexposed (no COVID‐19)	
	Median [q1–q3]	Mean (SD)	Median [q1–q3]	Mean (SD)
Periods before index date				
2018	3 [1–7]	5.6 (9.3)	2 [0–6]	5.3 (8.9)
2019	3 [1–7]	6.0 (10.0)	2 [0–7]	5.7 (9.6)
13–1 month pre‐index	3 [1–8]	6.2 (10.7)	2 [0–7]	5.6 (10.0)
Period after index date				
4–15 months post‐index	3 [1–8]	6.7 (12.0)	2 [0–7]	5.8 (10.8)

Abbreviation: SD, standard deviation.

### DID‐analysis of the impact of COVID‐19 on number of healthcare contacts

**Fig. 4:**
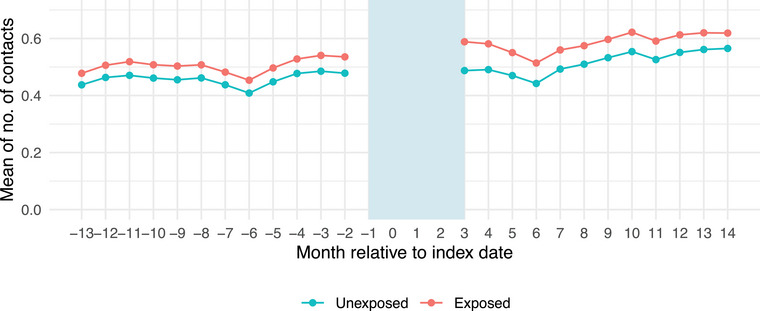
Trends in number of healthcare contacts before and after index date for the exposed (registered COVID‐19) and unexposed (no COVID‐19) group. The study population includes all adult residents in the two largest counties in Sweden in 2020.

The trends in number of healthcare contacts in the unexposed and exposed group appeared parallel before index date (Fig. 4). The DID analysis showed that the difference in the mean number of healthcare contacts between exposed and unexposed was 0.33 (95%CI 0.30, 0.36) healthcare contacts after COVID‐19 (Table 3). Overall, 84% of this mean value increase was attributed to increases in primary healthcare contacts. The 10 most frequent diagnosis categories with a statistically significantly DID difference included, for example, severe stress and malaise and fatigue (Table 4). Scaled to a population level, the DID estimate of 0.33 corresponds to approximately 33,000 additional healthcare contacts per 100,000 individuals diagnosed with COVID‐19 during the 12 months following 3 months from infection. Healthcare contacts increased substantially during the initial months of follow‐up but gradually returned to baseline levels over time (Fig. S4).

**Table 3:** Results from the difference‐in‐difference analysis.

Healthcare contact	Exposed (COVID‐19) (post–pre)	Unexposed (no COVID‐19) (post–pre)	DID (95%CI)^a^ Exposed–unexposed
Total	0.56	0.23	0.33 (0.30, 0.36)
Primary care telephone	0.13	0.07	0.06 (0.05, 0.07)
Primary care other	0.43	0.16	0.26 (0.24, 0.29)
Outpatient hospital specialist care	0.09	0.02	0.07 (0.06, 0.08)
Inpatient hospital specialist care	0.01	0.01	0.00 (0.00, 0.01)

*Note*: Mean difference in number of healthcare contacts the year after (4–15 months post‐index) versus before index date (13–1 months pre‐index) for exposed (registered COVID‐19) and unexposed (no COVID‐19) Individuals in a study population including all matched adult residents in the two largest counties in Sweden in 2020. The total number of healthcare contacts as well as categorized contacts according to level of healthcare are presented. Post period  =  4–15 months after index date. Pre period  =  13–1 month before index date.

Abbreviations: CI, confidence interval; DID, difference in difference.

^a^ Difference in difference analysis (mean value for the period after minus the period before for the exposed minus the mean value for the period after minus the period before for the unexposed).

**Table 4:** Top 10 diagnoses with largest difference‐in‐difference (DID).

ICD10‐SE code	Diagnosis description	Exposed (COVID‐19) (post–pre)	Unexposed (no COVID‐19) (post–pre)	DID^a^ (95%CI) Exposed–unexposed
F43	Reaction to severe stress, and adjustment disorders	0.010	−0.010	0.020 (0.012, 0.028)
O26	Maternal care for other conditions predominantly related to pregnancy	0.005	−0.015	0.020 (0.017, 0.023)
R53	Malaise and fatigue	0.018	0.003	0.015 (0.013, 0.016)
F41	Other anxiety disorders	0.009	−0.005	0.013 (0.008, 0.019)
G93^b^	Other disorders of brain	0.012	0.001	0.012 (0.010, 0.013)
R06	Abnormalities of breathing	0.011	0.003	0.008 (0.007, 0.010)
Z73	Problems related to life management difficulty	0.006	−0.001	0.007 (0.004, 0.010)
Z49	Encounter for care involving renal dialysis	0.007	0.000	0.007 (0.003, 0.011)
Z39	Encounter for maternal postpartum care and examination	0.004	−0.003	0.007 (0.006, 0.007)
J96	Respiratory failure, not elsewhere classified	0.007	0.001	0.006 (0.005, 0.008)

*Note*: Mean number of healthcare contacts comparing exposed (COVID‐19) individuals to their unexposed (no COVID‐19) matched individuals. *p*‐values adjusted by a false discovery rate of 0.05. The study population includes all adult residents in the two largest counties in Sweden in 2020.

Abbreviation: ICD‐10‐SE, International Classification of Diseases version 10 Swedish edition.

^a^Difference in difference analysis (mean value for the period after minus the period before for the exposed minus the mean value for the period after minus the period before for the unexposed).

^b^Mainly G93.3 Postural and related fatigue syndromes.

**Fig. 5:**
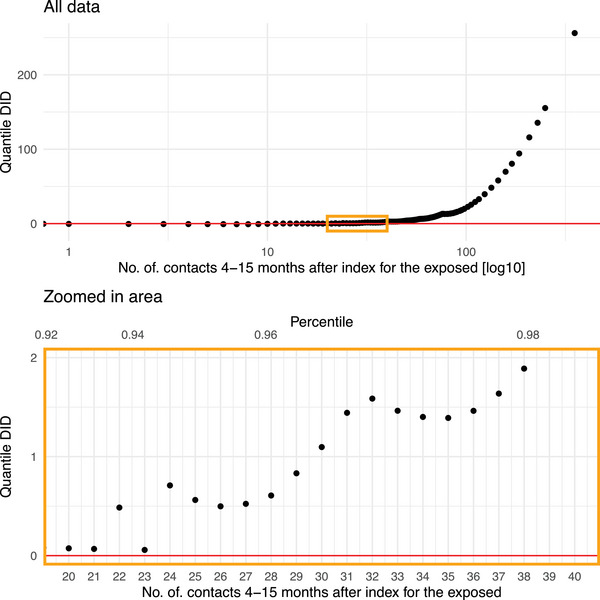
Quantile difference‐in‐differences (DID) estimates of the distributional impact of COVID‐19 exposure on the number of healthcare contacts 4–15 months after index among the exposed. Zoomed in area for where in the distribution the DID estimates start to increase. The study population includes all adult residents in the two largest counties in Sweden in 2020.

Despite the observed increase in the mean number of healthcare contacts, most individuals in both the unexposed and exposed groups had the same number of healthcare contacts before and after the index date (Fig. S5). A decrease in the number of healthcare contacts was equally common in both groups, whereas an increase was slightly more frequent among the exposed (Fig. S5). In the QDID‐analysis, an increase in the number of healthcare contacts was observed from the 95th percentile and above. This corresponds to individuals with at least 24 healthcare contacts during the post‐index period (4–15 months) and with an IQR of 7–30 in the pre‐index period (Fig. 5).

### Subgroup analyses

**Fig. 6:**
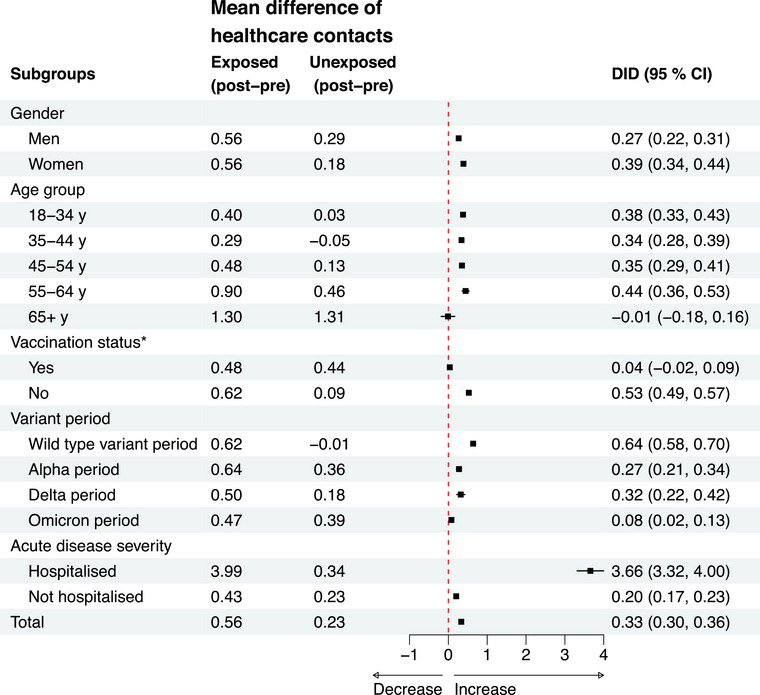
Forest plot of the conditional difference‐in‐difference (DID) estimates of the impact of COVID‐19 exposure on healthcare contacts by subgroups. The study population includes all adult residents in the two largest counties in Sweden in 2020. *Vaccination status before infection (yes  =  ≥1 dose, no  =  0 doses). CI, confidence interval.

The DID was higher in women than men (0.39 [0.34, 0.44] vs. 0.27 [0.22, 0.31]) (Fig. 6, Table S1). Similarly, individuals who were hospitalized due to COVID‐19 had a greater increase in healthcare contacts than those who were not hospitalized (3.66 [3.32, 4.00] vs. 0.20 [0.17, 0.23]) (Fig. 6, Table S2). Individuals who were vaccinated before index had lower DID (0.04 [−0.02, 0.09]) than those that were not vaccinated (0.53 [0.49, 0.57]) (Fig. 6, Table S3). The DID also varied by the dominant virus variant period, with the highest increase observed during the wild‐type variant period (0.64 [0.58, 0.70]) compared to the Alpha (0.27 [0.21, 0.34]), Delta (0.32 [0.22, 0.42]) and Omicron variant periods (0.08 [0.02, 0.13]) (Fig. 6, Table S4). The most frequent diagnosis codes for levels of care and strata are presented in Table S5.

### Sensitivity analysis

The DID estimate was higher (0.59 [0.55, 0.62] vs. 0.33 [0.30, 0.36]) when not ending follow‐up at reinfection for the exposed individuals, probably because counting contacts related to the acute reinfection resulted in inclusion of more contacts for the exposed individuals in the post‐period and a larger DID estimate.

## Discussion

This study uses a DID‐approach to compare exposed and unexposed individuals’ healthcare utilization before and after COVID‐19, while controlling for underlying trends in both groups. The findings indicate higher healthcare utilization following a COVID‐19 diagnosis, concentrated in approximately 5% of the population, primarily within primary healthcare, and with variation across subgroups. This study included information on all healthcare contacts at all levels of care in a large, comprehensive population (*n* = 1,415,432 unique individuals in the matched study sample) based on a source population of approximately 3 million individuals in the two largest counties in Sweden. Some of the main diagnoses associated with increased healthcare utilization were stress, anxiety and fatigue—symptoms that have been closely associated with post‐COVID‐19 conditions [23], indicating that post‐COVID‐19 conditions could be a major cause of the increased healthcare utilization after COVID‐19.

Some previous studies have evaluated the change in healthcare utilization before and after COVID‐19 [12, 24, 25, 26, 27], varying in study population, length of follow‐up, study design, disease severity and virus variants studied. A Norwegian register‐based study assessed the difference in healthcare utilization between test‐positive adults with mild COVID‐19 during the first year of the pandemic and test‐negative individuals. Using a DID‐approach, an elevated primary healthcare consumption for the test‐positive during the first 3 months directly after the acute infection comparing to 3 months before was seen, whereas no increase was observed after 4–6 months [12]. Another Norwegian study examined the change in healthcare utilization between 6 months before and 6 months after COVID‐19 among non‐hospitalized children compared to test‐negative and non‐tested children in a DID‐analysis, during the first year of the pandemic [24]. Results showed an increased utilization of primary healthcare during the first month for all age groups, which gradually declined over the following 6 months. A population‐based Danish cohort study, including SARS‐CoV‐2 test‐positive and test‐negative individuals tested during the first 3 months of the pandemic, showed increased number of primary healthcare visits in the test‐positive group compared to test‐negative controls the first 6 months after infection, with adjustment for prior healthcare visits [26]. An increased number of healthcare visits for adults over 40 years of age with COVID‐19 was also observed 6 months after infection in an Italian study comparing healthcare utilization after COVID‐19 with matched unexposed individuals [27]. In contrast to these studies, our analysis sharpens the focus on post‐COVID‐19 conditions by excluding the acute effects of COVID‐19 in the first 3 months, has longer follow‐up, and includes a larger part of the pandemic period, covering several virus variants. This contributes to the accumulating knowledge on how the long‐term effects after COVID‐19 also affect the healthcare system and society.

Our subgroup analyses showed that the DID estimate was higher for women than for men, for patients that were hospitalized for COVID‐19 during the acute phase of the infection than for non‐hospitalized, for unvaccinated than for vaccinated and for the wild‐type variant period than for later periods of virus variants. It has previously been shown that female sex is a risk factor for long‐term symptoms after COVID‐19 [28] and as women seek healthcare more often than men [29], it is not surprising that we found that women had a larger increase in healthcare utilization after COVID‐19 than men. Infections during the Omicron period did not increase healthcare utilization as much after infection, perhaps because the infection with the Omicron variant was milder and thus did not result in an increase in healthcare utilization or because there was less frequent SARS‐CoV‐2 testing in the population during the Omicron dominating period, making it more likely that individuals in the control group had COVID‐19 that was not identified which could tend to bias the DID estimate to the null due to misclassification of the exposure.

Scaled to a population level, the DID estimate corresponds to roughly 33,000 additional healthcare contacts per 100,000 individuals diagnosed with COVID‐19 during the 12 months following 3 months from infection. Although most individuals show no or only a small change in healthcare utilization, the aggregate effect on the population level implies a substantial increase in the total healthcare utilization. The increased healthcare utilization after COVID‐19 does not reflect a strong increase in healthcare consumption in the whole population as the increase was mainly observed for approximately 5% of the infected population, corresponding to individuals with 24 visits or more during the year after COVID‐19. There have been numerous reports on persistent long‐term symptoms after COVID‐19, so called post‐COVID‐19 conditions. The diagnosis codes associated with the observed increase in the present study were mainly symptoms and conditions that have been associated with post‐COVID‐19 conditions and are managed in primary healthcare, strengthening the hypothesis that the increase was at least partly driven by post‐COVID‐19 conditions. A prospective cohort study from Spain, evaluating the recovery from post‐COVID‐19 condition with 2‐years of follow‐up, showed that only a minority of patients (7.6%) recovered during this period [30], whereas others have shown better recovery rates after COVID‐19 [13]. The cost for patients with post‐COVID‐19 condition in United Kingdom has been estimated to be considerable, a 44% increased cost in primary healthcare consultation for patients reporting long‐term symptoms compared to patients who do not the first year after infection [31]. Our study shows that long‐term symptoms after COVID‐19 not only have an individual‐level effect but could also have a capacity to affect the healthcare system with an increase in the mean number of healthcare contacts. This could have implications for societal allocation of resources to the healthcare system and highlights that SARS‐CoV‐2 still needs to be taken into consideration when planning healthcare.

### Strengths and limitations

The strengths of the present large population‐based study include the following: (1) all adults with a registered COVID‐19 during the inclusion period from the two largest counties in Sweden were included, (2) several data sources from all levels of care—with near complete coverage—were used to obtain the most accurate estimates of healthcare contacts, (3) a DID analysis was employed, which controls for time‐invariant confounding, and exposed and unexposed groups showed parallel trends in healthcare utilization before the index date, (4) follow‐up began 3 months after the registered infection to focus on the potential long‐term effects of healthcare utilization after COVID‐19, and (5) the study period covered a large part of the pandemic, including several different dominant virus variants.

Limitations of the present study include that (1) not all individuals had complete follow‐up; for some, the number of healthcare contacts during a full year was estimated based on the observed number of contacts before termination of follow‐up. However, 91% of the exposed and 88% of unexposed individuals had a full year of follow‐up (15 months after index date), making substantial unidirectional bias unlikely. (2) Our study evaluated healthcare utilization after a first registered (primary) COVID‐19. Whether our results apply for today's population—most of whom have some level of immunity from previous infections and vaccinations—remains uncertain. (3) The unexposed group was defined by the absence of registered COVID‐19; therefore, it is possible that some individuals in this group were infected but never tested, potentially leading to misclassification and a biased DID estimate to the null.

## Conclusion

In an analysis of data spanning the full course of the COVID‐19 pandemic, we observe an increased mean number of healthcare contacts after COVID‐19, mainly observed for approximately 5% of the population, mainly in the primary healthcare setting, and with variation across subgroups. Stress, anxiety and fatigue were the most common diagnoses contributing to the increase. These findings are important for planning of healthcare in post‐pandemic scenarios and are helpful for better understanding of healthcare utilization after infectious diseases.

## Author contributions

Maria Bygdell, Erik Bülow, Jörgen Månsson, Kristoffer Strålin and Fredrik Nyberg conceived and designed the study; Erik Bülow and Jari Martikainen performed the statistical analysis; all authors interpreted the results; Maria Bygdell drafted the first draft of the manuscript; all authors revised the manuscript for important intellectual content and approved the final version to be submitted.

## Conflict of interest statement

Maria Bygdell, Erik Bülow, Simon B. Larsson, Robert Sigström, Huiqi Li, Jari Martikainen, Ailiana Santosa, Lisa Lundberg‐Morris, Carl Bonander, Jörgen Månsson and Kristoffer Strålin declare no conflicts of interest. Susannah Leach is an employee of AstraZeneca. Magnus Gisslén received research grants from Gilead Sciences and honoraria as a speaker, DSMB committee member and/or scientific advisor from Amgen, AstraZeneca, Biogen, Bristol‐Myers Squibb, Gilead Sciences, GlaxoSmithKline/ViiV, Janssen‐Cilag, MSD, Novocure, Novo Nordic, Pfizer and Sanofi (outside of submitted work). All mentioned engagements have concluded and are not ongoing. Fredrik Nyberg owns some AstraZeneca shares.

## Funding information

Maria Bygdell has funding grants from the Swedish Society for Medical Research (PD20‐0012); the Swedish Research Council for Health, Working Life and Welfare (2022‐00444); and the Swedish Research Council (2022‐06395). Lisa Lundberg‐Morris has funding grants from the Swedish state under the agreement between the Swedish government and the county councils, the ALF agreement (ALFGBG‐997083). Magnus Gisslén has funding grants from the Swedish state, under an agreement between the Swedish government and the county councils (ALF agreement ALFGBG‐1005848), the Swedish Research Council (2021‐05045 and 2021‐06545) and King Gustaf V's and Queen Victoria's Foundation. Carl Bonander has funding grants from the Swedish Research Council for Health, Working Life and Welfare (2020‐00962, 2023‐01104). Fredrik Nyberg received funding from SciLifeLab from the Knut and Alice Wallenberg Foundation (KAW 2021‐0010/VC2021.0018 and KAW 2020.0299/VC 2022.0008) and the Swedish Research Council (2021‐05045 and 2021‐05450). Additionally, the SCIFI‐PEARL (Swedish Covid‐19 Investigation for Future Insights—a Population Epidemiology Approach using Register Linkage) project, which provides the data for this analysis, has basic funding through Fredrik Nyberg based on grants from the Swedish state under the agreement between the Swedish government and the county councils, the ALF‐agreement (ALFGBG‐938453, ALFGBG‐971130, ALFGBG‐978954 and ALFGBG‐1006729), a grant from the Swedish Research Council for Health, Working Life and Welfare (2024‐01711) and previously from a joint grant from the Swedish Research Council for Health, Working Life and Welfare and Swedish Research Council for Environment, Agricultural Sciences and Spatial Planning (2020‐02828).

## Supporting information




**Supporting File 1**: joim70051‐sup‐0001‐SuppMat.docx

## Data Availability

The data used in this study are pseudonymized individual‐level data from Swedish healthcare registers and can be obtained from the respective Swedish public data holders on the basis of ethics approval for the research in question, subject to relevant legislation, processes and data protection.
