# Supplementary material for: Change in healthcare utilization before and after COVID‐19 using data from 1.5 million individuals
Source: J Intern Med. 2025 Nov 27;299(2):255–70. doi: 10.1111/joim.70051 (PMC12789290; doi:10.1111/joim.70051)
Supplement: Supplementary file 1 — Supporting File 1: joim70051‐sup‐0001‐SuppMat.docx [file JOIM-299-255-s001.docx]

**Supplemental material**

**Healthcare utilisation before and after COVID-19 using data from 1.5 million individuals**

Maria Bygdell, Erik Bülow, Simon B Larsson, Robert Sigström, Huiqi Li, Jari Martikainen, Ailiana Santosa, Lisa Lundberg-Morris, Susannah Leach, Magnus Gisslén, Carl Bonander, Jörgen Månsson, Kristoffer Strålin*, Fredrik Nyberg*

**Supplemental methods**

**Matching**

We aimed to estimate the average effect of having COVID-19 on healthcare utilisation among individuals who got a registered COVID-19 compared to not having a registered COVID-19, and since this is anchored on the group with COVID-19 and their time of infection, this is analogous to the average treatment effect among the treated (ATT). Since all individuals in the cohort were at risk of infection, their exposure status was time-dependent. Defining infection status as “ever infected” (i.e., considering future infections) would introduce non-intuitive estimands and risk of immortal time bias. To address this, we applied a risk-set matching or time-dependent exposure matching, following the approach described by Li et al. (Li YP, Propert KJ, Rosenbaum PR. Balanced Risk Set Matching. Journal of the American Statistical Association 2001; 96: 870–82). Matching was performed using the R package “heaven” (Thomas A. Gerds et al., https://github.com/tagteam/heaven), with slight modifications to the source code to enable matching without replacement among non-infected controls. We preferred to have unique controls, as the large pool of not yet infected individuals allowed us to achieve good covariate balance, reduce the dependency between matched pairs, and include all cases without the need to reuse controls. Individuals who later became infected, however, could also serve as matched controls prior to their recorded infection. Exact matching variables included year of birth, gender, change in number of healthcare contacts between 2018 and 2019, and vaccination status (vaccinated or not), with the latter also treated as a time-dependent variable. In addition, we performed three sensitivity analyses: 1) 1:1 matching with replacement, 2) 1:2 matching without replacement, and 3) 1:2 matching with replacement, resulting in very similar DID estimates (Supplemental Table S6)

**Incomplete follow-up**

Individuals were followed until termination of follow-up due to registered COVID-19 30 days later (to avoid including excess healthcare utilization leading up to the confirmed diagnosis itself), death, emigration, or move out of the included counties. The total proportion ending follow-up during 15 months after index date was 9 % of the exposed and 12 % of the unexposed (Supplemental Figures S1 and S2). The main reason for termination of follow-up among the unexposed was due to registered COVID-19 (in analogy with a per-protocol treatment strategy). Likewise, exposed individuals terminated the follow-up at registered re-infections, but this was less common. A smaller fraction terminated follow-up due to death, emigration, or move out of the included counties, whichever came first (Supplemental Table S7). There was no termination of follow-up due to unobserved outcome data (outcome data were available until 31 May 2024, which occurred more than 15 months after the last index date).

**Number of healthcare contacts**

The observed number of healthcare contacts was used for the pre-index period, as well as for individuals without termination of follow-up during the post-index period (91 % and 88 % of the exposed and unexposed individuals, respectively). If termination of follow-up occurred before the actual follow-up period started (within 0 to 90 days after the index date) for either the exposed or unexposed, the whole pair were excluded from the analysis. Individuals with less than 3 months of follow-up (termination of follow-up within 91 to 182 days from their index date) retained their observed number of healthcare contacts in their post-index period (4 to 15 months), as a conservative estimate of their actual healthcare consumption during the full year (since we did not want to extrapolate on a too short period). For individuals ending follow-up after at least 3 months of follow-up (within 270 to 450 days after their index date), their expected number of contacts for a full year follow-up was estimated by dividing the observed number by the fraction of time the individual was followed before termination (rounded to a whole number). This should give an unbiased estimate assuming that the accumulated number of healthcare contacts was constant during the follow-up period, which was confirmed for the observed contacts (Supplemental Figure S6). In most of the estimated cases (just over 50%), this adjustment added only one or two additional contacts to the observed count. To assess the potential impact of this estimation, we conducted a series of sensitivity analyses: 1) using only the observed number of contacts (ignoring possible contacts after termination of follow-up), which resulted in an unchanged overall mean difference-in-difference estimate (DID), 2) multiplied the extrapolation of all individuals without complete follow-up with factors ranging from 1-2, which resulted in a slightly higher DID (indicating that we have used a conservative approach), 3) multiplied the extrapolation of the exposed individuals with factors ranging from 1-2, which resulted in higher DID (indicating that we have used a conservative approach), and 4) multiplied the extrapolation of the unexposed individuals with factors ranging from 1-2, which resulted in a lower DID (indicating that we have used an optimistic approach). However, we do not think it is likely that the small proportion of unexposed individuals that we extrapolate would suddenly increase their number of healthcare contacts with a factor of for example 1.5 while the exposed individuals do not.

**Pair-specific differences and DID calculation**

Given a matched pair with one exposed (*E*) and one unexposed (*U*) individual, we calculated the difference in the number of observed (or estimated for some individuals) healthcare contacts as the number during the post-index period (*E_post_* and *U_post_*) minus the pre-index period (*E_pre_* and *U_pre_*). Hence, for each individual: *E_post_* – *E_pre_* and *U_post_* – *E_pre,_* respectively*_,_* and then for each pair, *DID = (E_post_ – E_pre_) – (U_post_ – E_pre_)*. The null hypothesis of a mean value of *DID at zero*, interpreted as equal differences in the number of healthcare contacts among the exposed and unexposed individuals between the post- and pre-index periods, was assessed by a one-group Student’s t-test. In addition to the overall mean DID, the estimation was performed stratified by level of care. For each level of care, we defined its contribution to the overall DID as the proportion of matched pairs with non-zero DID values within that care setting, multiplied by the overall average DID, and then normalized the resulting values across all care types so that the total sum was one, as matched pairs may contribute to more than one care type.

**Accounting for dependency**

It should be noted that calculating the paired DID-value as described above is equivalent to estimating the interaction term from a linear regression model with exposure status and period as main effects—an approach that is also commonly assessed using a t-test. Given the paired data, the dependency introduced by repeated measurements within individuals was addressed by reducing the individual time-dependent measurements to the difference between the two periods, which captured most of the dependency introduced by the matching algorithm. However, our exposure-matching strategy allowed some individuals, who later became exposed, to first serve as unexposed matches in additional pairs with an earlier index date. To account for this remaining dependency, we applied a post-test adjustment of standard errors (and consequently confidence intervals and p-values) using a heteroscedasticity-consistent (sandwich) covariance matrix estimation from a simple intercept-only linear regression model of the mean. As a result, our simplified use of a t-test should yield results equivalent to those obtained from a linear interaction model with robust standard errors. Given the large sample size, however, the robustness adjustment only had a minor effect on the estimated p-values (at most on the third decimal), and did not change the interpretation of statistical significance (p-value threshold of 0.05). One reason for preferring the t-test was computational efficiency, since the analysis was repeated for different diagnosis codes, and the robustness adjustment was only relevant for the few codes with initial statistical significance (adjusted for a false discovery rate of 0.05).

**Quantile DID**

We also assessed the distributional effects by estimating the Quantile Treatment Effect on the Treated (QTET)—or more precisely, the effect of being recorded as exposed among the exposed—by comparing the counterfactual distribution of the outcome in the exposed group to its observed post-treatment distribution. This comparison is valid under the assumptions of conditional distributional DID (a generalization of the parallel trends assumption, as illustrated in Figure 4 and further motivated by Supplementary Figure S6) and copula invariance (evaluated in a subsample of 10,000 observations).

**R-packages**

We used R version 4.4.1 (R Core Team 2024) and the following R packages: arrow v. 18.1.0.1 (Richardson et al. 2025), BMisc v. 1.4.8 (Callaway 2025), broom.mixed v. 0.2.9.6 (Bolker and Robinson 2024), coin v. 1.4.3 (Hothorn et al. 2006, 2008), copula v. 1.1.5 (Jun Yan 2007; Ivan Kojadinovic and Jun Yan 2010; Marius Hofert and Martin Mächler 2011; Hofert et al. 2025), cowplot v. 1.1.3 (Wilke 2024), data.table v. 1.16.4 (Barrett et al. 2024), decoder v. 1.2.2 (Bulow 2020), devtools v. 2.4.5 (Wickham et al. 2022), doParallel v. 1.0.17 (Corporation and Weston 2022), duckdb v. 1.1.3.2 (Mühleisen and Raasveldt 2025), effects v. 4.2.2 (Fox 2003; Fox and Hong 2009; Fox and Weisberg 2018, 2019), emmeans v. 1.10.6 (Lenth 2024), forestploter v. 1.1.2 (Dayimu 2024), formula.tools v. 1.7.1 (Brown 2018), fs v. 1.6.5 (Hester, Wickham, and Csárdi 2024), furrr v. 0.3.1 (Vaughan and Dancho 2022), future v. 1.34.0 (Bengtsson 2021), ggflowchart v. 1.0.0 (Rennie 2023), ggrepel v. 0.9.6 (Slowikowski 2024), ggsankey v. 0.0.99999 (D. Sjoberg 2024), glue v. 1.8.0 (Hester and Bryan 2024), grid v. 4.4.1 (R Core Team 2024b), gt v. 0.11.1 (Iannone et al. 2024), gtsummary v. 2.0.4 (D. D. Sjoberg et al. 2021), heaven v. 2022.7.13 (Rytgaard et al. 2024), here v. 1.0.1 (Müller 2020), knitr v. 1.49 (Xie 2014, 2015, 2024), lmtest v. 0.9.40 (Zeileis and Hothorn 2002), msm v. 1.8.2 (Christopher H. Jackson 2011), nanoparquet v. 0.3.1 (Csárdi and Mühleisen 2024), patchwork v. 1.3.0 (Pedersen 2024), pkgdown v. 2.1.1 (Wickham et al. 2024), progressr v. 0.15.1 (Bengtsson 2024), ProjectTemplate v. 0.11.0 (White 2024), pscl v. 1.5.9 (Zeileis, Kleiber, and Jackman 2008; Jackman 2024), qs v. 0.27.2 (Ching 2024), qte v. 1.3.1 (Callaway 2022), quantreg v. 5.99.1 (Koenker 2024), rmarkdown v. 2.29 (Xie, Allaire, and Grolemund 2018; Xie, Dervieux, and Riederer 2020; Allaire et al. 2024), rstatix v. 0.7.2 (Kassambara 2023), sandwich v. 3.1.1 (Zeileis 2004, 2006; Zeileis, Köll, and Graham 2020), scales v. 1.3.0 (Wickham, Pedersen, and Seidel 2023), stringfish v. 0.16.0 (Ching 2023), survival v. 3.8.3 (Terry M. Therneau and Patricia M. Grambsch 2000; Therneau 2024), survminer v. 0.5.0 (Kassambara, Kosinski, and Biecek 2024), texreg v. 1.39.4 (Leifeld 2013), tidyverse v. 2.0.0 (Wickham et al. 2019), tinytest v. 1.4.1 (van der Loo 2020), writexl v. 1.5.1 (Ooms 2024).

**Supplemental Tables**

**Table S1.** Median (q1-q3) and mean (SD) number of healthcare contacts in the different time periods in the exposed (COVID-19) and unexposed (no COVID-19) groups, by gender. The study population includes adult residents in the two largest counties in Sweden in 2020.

|  | **Exposed** | | **Unexposed** | |
| --- | --- | --- | --- | --- |
|  | Median (q1-q3) | Mean (SD) | Median (q1-q3) | Mean (SD) |
| **Periods before index date** | | | | |
| **2018** |  |  |  |  |
| Women | 4 (1-9) | 6.9 (10.1) | 3 (1-8) | 6.5 (9.8) |
| Men | 2 (0-5) | 4.2 (8.0) | 1 (0-5) | 3.9 (7.5) |
| **2019** |  |  |  |  |
| Women | 4 (1-9) | 7.4 (10.9) | 3 (1-9) | 7.0 (10.5) |
| Men | 2 (0-5) | 4.5 (8.8) | 2 (0-5) | 4.2 (8.2) |
| **13-1 month** |  |  |  |  |
| Women | 4 (1-9) | 7.4 (11.4) | 3 (1-9) | 6.8 (10.8) |
| Men | 2 (0-6) | 4.7 (9.6) | 1 (0-5) | 4.2 (8.6) |
| **Period after index date** | | | | |
| **4-15 months** |  |  |  |  |
| Women | 4 (1-10) | 8.0 (12.6) | 3 (1-9) | 7.0 (11.7) |
| Men | 2 (0-6) | 5.3 (11.1) | 1 (0-5) | 4.5 (9.6) |

**Table S2.** Median (q1-q3) and mean (SD) number of healthcare contacts in the different time periods in the exposed (COVID-19) and unexposed (no COVID-19) groups, by acute COVID-19 disease severity of the exposed individual and their individually matched* controls. The study population includes adult residents in the two largest counties in Sweden in 2020.

|  | **Exposed** | | **Unexposed** | |
| --- | --- | --- | --- | --- |
|  | Median (q1-q3) | Mean (SD) | Median (q1-q3) | Mean (SD) |
| **Periods before index date** | | | | |
| **2018** |  |  |  |  |
| Hospitalised | 6 (2-15) | 11.5 (17.1) | 4 (1-11) | 8.6 (12.8) |
| Non-hospitalised | 2 (0-7) | 5.4 (8.8) | 2 (0-6) | 5.2 (8.7) |
| **2019** |  |  |  |  |
| Hospitalised | 7 (2-16) | 12.9 (19.8) | 5 (1-12) | 9.9 (15.7) |
| Non-hospitalised | 3 (1-7) | 5.8 (9.4) | 2 (0-7) | 5.5 (9.2) |
| **13-1 month** |  |  |  |  |
| Hospitalised | 7 (2-17) | 13.9 (22.4) | 4 (1-11) | 9.2 (16.3) |
| Non-hospitalised | 3 (0-7) | 5.9 (9.9) | 2 (0-7) | 5.5 (9.6) |
| **Period after index date** | | | | |
| **4-15 months** |  |  |  |  |
| Hospitalised | 9 (3-21) | 17.9 (29.1) | 4 (1-11) | 9.5 (17.9) |
| Non-hospitalised | 3 (1-8) | 6.3 (10.6) | 2 (0-7) | 5.7 (10.4) |

* Matched for age, gender, vaccination status before index, and the change in number of healthcare contacts between 2018 and 2019.

**Table S3.** Median (q1-q3) and mean (SD) number of healthcare contacts in the different time periods in the exposed (COVID-19) and unexposed (no COVID-19) groups, by vaccination status before index. The study population includes adult residents in the two largest counties in Sweden in 2020.

|  | **Exposed** | | **Unexposed** | |
| --- | --- | --- | --- | --- |
|  | Median (q1-q3) | Mean (SD) | Median (q1-q3) | Mean (SD) |
| **Periods before index date** | | | | |
| **2018** |  |  |  |  |
| Vaccinated | 2 (0-7) | 5.4 (8.9) | 2 (0-6) | 5.2 (8.9) |
| Not vaccinated | 3 (1-7) | 5.8 (9.5) | 2 (0-7) | 5.3 (8.9) |
| **2019** |  |  |  |  |
| Vaccinated | 3 (1-7) | 5.8 (9.6) | 2 (0-7) | 5.7 (9.6) |
| Not vaccinated | 3 (1-8) | 6.2 (10.3) | 2 (0-7) | 5.7 (9.6) |
| **13-1 month** |  |  |  |  |
| Vaccinated | 3 (1-8) | 6.2 (11.0) | 2 (0-7) | 5.7 (10.3) |
| Not vaccinated | 3 (1-8) | 6.2 (10.5) | 2 (0-7) | 5.5 (9.7) |
| **Period after index date** | | | | |
| **4-15 months** |  |  |  |  |
| Vaccinated | 3 (1-8) | 6.6 (11.8) | 3 (0-7) | 6.2 (11.2) |
| Not vaccinated | 3 (0-8) | 6.8 (12.1) | 2 (0-7) | 5.6 (10.6) |

**Table S4.** Median (q1-q3) and mean (SD) number of healthcare contacts in the different time periods in the exposed (COVID-19) and unexposed (no COVID-19) groups, by time periods of predominant virus variant for the index date in COVID-19 exposed and their individually matched* controls. The study population includes adult residents in the two largest counties in Sweden in 2020.

|  | **Exposed** | | **Unexposed** | |
| --- | --- | --- | --- | --- |
|  | Median (q1-q3) | Mean (SD) | Median (q1-q3) | Mean (SD) |
| **Periods before index date** | | | | |
| **2018** |  |  |  |  |
| Wild type variant | 3 (1-8) | 6.5 (10.5) | 3 (0-7) | 5.8 (9.5) |
| Alpha variant | 2 (0-6) | 5.1 (8.4) | 2 (0-6) | 5.0 (8.4) |
| Delta variant | 2 (0-6) | 5.3 (8.8) | 2 (0-6) | 5.1 (8.7) |
| Omicron variant | 2 (0-6) | 5.2 (8.7) | 2 (0-6) | 5.0 (8.6) |
| **2019** |  |  |  |  |
| Wild type variant | 3 (1-9) | 7.0 (11.4) | 3 (1-8) | 6.3 (10.4) |
| Alpha variant | 2 (0-7) | 5.5 (9.0) | 2 (0-7) | 5.4 (9.0) |
| Delta variant | 2 (0-7) | 5.6 (9.3) | 2 (0-7) | 5.4 (9.3) |
| Omicron variant | 2 (0-7) | 5.6 (9.5) | 2 (0-7) | 5.4 (9.3) |
| **13-1 month** |  |  |  |  |
| Wild type variant | 3 (1-9) | 6.9 (11.6) | 3 (0-7) | 6.1 (10.5) |
| Alpha variant | 2 (0-7) | 5.4 (9.4) | 2 (0-6) | 5.1 (9.3) |
| Delta variant | 3 (0-7) | 6.0 (10.2) | 2 (0-7) | 5.5 (10.1) |
| Omicron variant | 3 (0-7) | 6.0 (10.6) | 2 (0-7) | 5.5 (9.8) |
| **Period after index date** | | | | |
| **4-15 months** |  |  |  |  |
| Wild type variant | 3 (1-9) | 7.6 (13.4) | 2 (0-7) | 6.0 (11.5) |
| Alpha variant | 3 (0-7) | 6.1 (10.6) | 2 (0-7) | 5.5 (10.1) |
| Delta variant | 3 (1-8) | 6.5 (11.6) | 2 (0-7) | 5.6 (10.8) |
| Omicron variant | 3 (1-8) | 6.4 (11.5) | 2 (0-7) | - 1. (10.6) |

* Matched for age, gender, vaccination status before index, and the change in number of healthcare contacts between 2018 and 2019.

**Table S5.** The three most frequent disease diagnosis categories, by strata. The study population includes adult residents in the two largest counties in Sweden in 2020.

| **Strata** | **ICD10-SE code** | **Diagnosis description** | **Exposed**  **(COVID-19)**  (post - pre) | **Unexposed**  **(no COVID-19)**  (post - pre) | **DID* (95%CI)**  Exposed - unexposed |
| --- | --- | --- | --- | --- | --- |
| **Level of care** |  |  |  |  |  |
| Primary care telephone (n=827,592**) | R53 | Malaise and fatigue | 0.004 | 0.001 | 0.004, (0.003, 0.004) |
|  | G93*** | Other disorders of brain | 0.002 | -0.001 | 0.002, (0.002, 0.003) |
|  | O26 | Maternal care for other conditions predominantly related to pregnancy | -0.002 | -0.004 | 0.002, (0.002, 0.003) |
| Primary care other (n=1,232,392**) | O26 | Maternal care for other conditions predominantly related to pregnancy | 0.007 | -0.011 | 0.017, (0.014, 0.020) |
|  | F43 | Reaction to severe stress, and adjustment disorders | 0.008 | -0.009 | 0.017, (0.010, 0.024) |
|  | R53 | Malaise and fatigue | 0.014 | 0.002 | 0.012, (0.010, 0.013) |
| Outpatient hospital specialist care (n=857,384**) | N97 | Female infertility | -0.001 | -0.004 | 0.003, (0.002, 0.004) |
|  | Z34 | Encounter for supervision of normal pregnancy | 0.000 | -0.003 | 0.003, (0.002, 0.003) |
|  | F90 | Attention-deficit hyperactivity disorders | 0.001 | -0.001 | 0.002, (0.001, 0.003) |
| Inpatient hospital specialist care (n=199,764**) | O80 | Encounter for full-term uncomplicated delivery | 0.002 | -0.003 | 0.005, (0.005, 0.006) |
|  | O82 | Encounter for cesarean delivery without indication | 0.001 | -0.001 | 0.001, (0.001, 0.002) |
|  | K80 | Cholelithiasis | 0.000 | -0.000 | 0.000, (0.000, 0.001) |
| **Gender** |  |  |  |  |  |
| Men (n=700,270) | F41 | Other anxiety disorders | 0.004 | -0.002 | 0.006, (0.003, 0.009) |
|  | R53 | Malaise and fatigue | 0.006 | 0.001 | 0.005, (0.004, 0.006) |
|  | G93*** | Other disorders of brain | 0.005 | 0.000 | 0.005, (0.004, 0.006) |
| Women (n=807,540) | O26 | Maternal care for other conditions predominantly related to pregnancy | 0.005 | -0.015 | 0.020, (0.017, 0.023) |
|  | F43 | Reaction to severe stress, and adjustment disorders | 0.008 | -0.008 | 0.016, (0.009, 0.024) |
|  | R53 | Malaise and fatigue | 0.011 | 0.002 | 0.009, (0.008, 0.011) |
| **Acute disease severity** |  |  |  |  |  |
| Hospitalised (n=28,153) | E11 | Type 2 diabetes mellitus | 0.013 | 0.003 | 0.010 (0.006, 0.015) |
|  | I50 | Heart failure | 0.007 | 0.001 | 0.006 (0.004, 0.009) |
|  | Z73 | Problems related to life management difficulty | 0.004 | -0.001 | 0.005, (0.004, 0.007) |
| Not hospitalised (n=725,752) | O26 | Maternal care for other conditions predominantly related to pregnancy | 0.007 | -0.015 | 0.022, (0.018, 0.025) |
|  | F43 | Reaction to severe stress, and adjustment disorders | 0.009 | -0.008 | 0.018, (0.010, 0.026) |
|  | R53 | Malaise and fatigue | 0.015 | 0.003 | 0.012, (0.010, 0.014) |

ICD-10-SE=International Classification of Diseases version 10 Swedish edition

* Difference in difference analysis (mean value for the period after minus the period before for the exposed minus the mean value for the period after minus the period before for the unexposed)

** Number of individuals with at least one contact in each level of care in pre- and post-periods

*** Mainly G93.3 Postural and related fatigue syndromes

**Table S6.** DID estimates from different matching strategies. The study population originates from adult residents in the two largest counties in Sweden in 2020.

| **Matching strategy** | **DID*** | **p-value** | **Exposed**  **n** | **Unexposed**  **n** | **Unique unexposed**  **n** |
| --- | --- | --- | --- | --- | --- |
| 1:1 without replacement** | 0.33 | <0.001 | 753,905 | 753,905 | 753,905 |
| 1:1 with replacement | 0.31 | <0.001 | 757,879 | 757,879 | 632,825 |
| 1:2 without replacement | 0.37 | <0.001 | 776,052 | 1,481,830 | 1,481,830 |
| 1:2 with replacement | 0.31 | <0.001 | 783,022 | 1,515,746 | 1,076,311 |

* Difference in difference analysis (mean value for the period after minus the period before for the exposed minus the mean value for the period after minus the period before for the unexposed)

** The matching strategy we use in the main analysis.

**Table S7.** Cumulative number of reasons for termination of follow-up, by exposure status. The study population includes adult residents in the two largest counties in Sweden in 2020.

|  | **Infection** | **Death** | **Emigration** | **Moved out of counties** |
| --- | --- | --- | --- | --- |
| Exposed  (COVID-19) | 60,252 (7.99%) | 2481 (0.33%) | 737 (0.10%) | 2117 (0.28%) |
| Unexposed  (no COVID-19) | 83,063 (11.02%) | 1671 (0.22%) | 1699 (0.23%) | 2054 (0.27%) |

**Supplemental figures**

**Figure S1.** Flow chart of the inclusion of the study population including all adult residents in the two largest counties in Sweden in 2020. Index date was set to the date of the registered COVID-19 for the exposed individuals and a risk set matching was used to create an unexposed comparison group matched 1:1 on birth year, gender, vaccination status before index, and the change in number of healthcare contacts between the two pre-index years 2018 and 2019. Individuals in the unexposed group had no registered COVID-19 up until the index date of their matched COVID-19 exposed counterpart.

**Figure S2.** Kaplan-Meier curve illustrating time until termination of follow-up after matching. The study population includes adult residents in the two largest counties in Sweden in 2020.

**Figure S3:** Sankey plots illustrating individual changes in number of healthcare contacts before versus after index date for the exposed (registered COVID-19) and unexposed (no COVID-19) groups. The study population includes adult residents in the two largest counties in Sweden in 2020.

**Figure S4.** Trend in the difference-in-difference (DID) estimate (95%CI based on 200 bootstrap replicates) before and after index date by month. The DID estimate each month was calculated as the difference between exposed and unexposed groups’ monthly deviation in the post-period estimates compared to the overall mean for the pre-period (by exposure status) The study population includes adult residents in the two largest counties in Sweden in 2020.

**Figure S5:** Difference between the number of healthcare contacts before and after index date in the exposed (registered COVID-19) and unexposed (no COVID-19) group. Note that the DID curve accounts for differences within matched pairs and is therefore not simply the difference between the two other curves. The grey line belongs to the upper x-axis and the black line to the lower x-axis. The study population includes all adult residents in the two largest counties in Sweden in 2020.

**Figure S6.** Number of observed healthcare contacts accumulated over time during 13 to 1 months before index and 4 to 15 months after index for exposed and unexposed individuals by level of care. The study population includes all adult residents in the two largest counties in Sweden in 2020.
